# Midnolin is a novel regulator of parkin expression and is associated with Parkinson’s Disease

**DOI:** 10.1038/s41598-017-05456-0

**Published:** 2017-07-19

**Authors:** Yutaro Obara, Toru Imai, Hidenori Sato, Yuji Takeda, Takeo Kato, Kuniaki Ishii

**Affiliations:** 1 Department of Pharmacology, Yamagata University School of Medicine, 2-2-2 Iida-Nishi, Yamagata, 990-9585 Japan; 2 Genome Informatics Unit, Institution for Promotion of Medical Science Research, Yamagata University School of Medicine, 2-2-2 Iida-Nishi, Yamagata, 990-9585 Japan; 3 Department of Immunology, Yamagata University School of Medicine, 2-2-2 Iida-Nishi, Yamagata, 990-9585 Japan; 4 Department of Neurology, Hematology, Metabolism, Endocrinology and Diabetology, Yamagata University School of Medicine, 2-2-2 Iida-Nishi, Yamagata, 990-9585 Japan

## Abstract

Midnolin (MIDN) was first discovered in embryonic stem cells, but its physiological and pathological roles are, to date, poorly understood. In the present study, we therefore examined the role of MIDN in detail. We found that in PC12 cells, a model of neuronal cells, MIDN localized primarily to the nucleus and intracellular membranes. Nerve growth factor promoted MIDN gene expression, which was attenuated by specific inhibitors of extracellular signal-regulated kinases 1/2 and 5. MIDN-deficient PC12 cells created using CRISPR/Cas9 technology displayed significantly impaired neurite outgrowth. Interestingly, a genetic approach revealed that 10.5% of patients with sporadic Parkinson’s disease (PD) had a lower MIDN gene copy number whereas no copy number variation was observed in healthy people, suggesting that MIDN is involved in PD pathogenesis. Furthermore, the expression of parkin, a major causative gene in PD, was significantly reduced by CRISPR/Cas9 knockout and siRNA knockdown of MIDN. Activating transcription factor 4 (ATF4) was also down-regulated, which binds to the cAMP response element (CRE) in the parkin core promoter region. The activity of CRE was reduced following MIDN loss. Overall, our data suggests that MIDN promotes the expression of parkin E3 ubiquitin ligase, and that MIDN loss can trigger PD-related pathogenic mechanisms.

## Introduction

Midbrain nucleolar protein (midnolin, MIDN) was first discovered by Tsukahara *et al*. using a gene trap approach in embryonic stem cells^1^. They reported that MIDN was strongly expressed in the midbrain of day E12.5 mice, and experiments with green fluorescent protein (GFP) tagged-MIDN showed that the protein localized primarily to the nucleus and nucleolus via a nucleolar localizing signal located in its C-terminal region. Furthermore, MIDN contains a ubiquitin-like domain at the N-terminal region. They showed that MIDN mRNA was strongly and abundantly expressed during early developmental stages, and that the expression was not limited to midbrain but also expressed in diverse adult mouse tissues, including heart, spleen, lung, liver, skeletal muscle, kidney and testis^1^. Subsequent microarray and reverse transcription-real-time polymerase chain reaction (RT-PCR) experiments revealed that MIDN acts as a transcription factor, controlling development through the regulation of mRNA transport^2^. Then, many years after its discovery, MIDN was found to interact with glucokinase via an N-terminal ubiquitin-like domain with high homology to parkin ubiquitin E3 ligase, leading to a significant reduction in the glucokinase activity, and glucose-induced insulin secretion from MIN6 cells^3^. In contrast to the study of the first group, this group observed the intracellular localization of MIDN was not in nucleoli but in nucleus and cytoplasm in pancreatic β-cells^3^. Overall, while MIDN was discovered many years ago, it is relatively poorly studied, and its physiological and pathological roles remain unclear.

Parkinson’s disease (PD) is a serious neurodegenerative disease involving the selective degeneration of dopaminergic neurons of the substantia nigra region of the midbrain which project into the striatum, resulting in a progressive extrapyramidal disorder. In approximately 10% of PD patients, the disease is familial, and many causative genes have been identified including α-synuclein, parkin, PTEN-induced putative kinase 1, DJ-1, and the leucine repeat-rich kinases^4, 5^. Such causative genes may be autosomal dominant or autosomal recessive, and their pathogenic mechanism may or may not include the deposition of Lewy bodies. In the other 90% of PD patients, however, the disease occurs sporadically and its causes are poorly understood, and so further research is required.

Previously, we reported that extracellular signal-regulated kinase 5 (ERK5), a member of mitogen-activated protein kinase (MAPKs), regulated neuronal functions in PC12 cells which are a model of dopaminergic cells and primary cultured sympathetic neurons. For examples, ERK5 was responsible for neurite outgrowth in PC12 cells and axon elongation in sympathetic neurons^6, 7^. In addition, the expression of tyrosine hydroxylase (TH) which is a rate-limiting enzyme for catecholamine biosynthesis was dramatically down-regulated by ERK5 knockdown in both cells, resulting in reduction in catecholamine biosynthesis. Furthermore, ERK5 and TH protein expression levels were significantly correlated in normal human adrenal medullas^7^. We therefore consider ERK5 to be a critical signaling molecule in catecholamine-secreting neurons and associated with catecholamine-related diseases such as PD. We have carried out the microarray screening to discover the genes that are regulated by ERK5 signaling^7^. We found that MIDN gene expression was upregulated by nerve growth factor (NGF), which was blocked by ERK5 inhibitor. Because physiological roles of MIDN are poorly understood as described above, we sought to clarify the roles of MIDN in neuronal cells. We discovered that 10.5% of patients with sporadic PD displayed a decreased MIDN gene copy number, whereas healthy participants showed no loss of MIDN. We then examined the physiological and pathological roles of MIDN in neuronal cells, as well as the pathological mechanisms of MIDN depletion in PD.

## Results

### MIDN induction by NGF is mediated via ERK1/2 and ERK5 in PC12 cells

**Figure 1:**
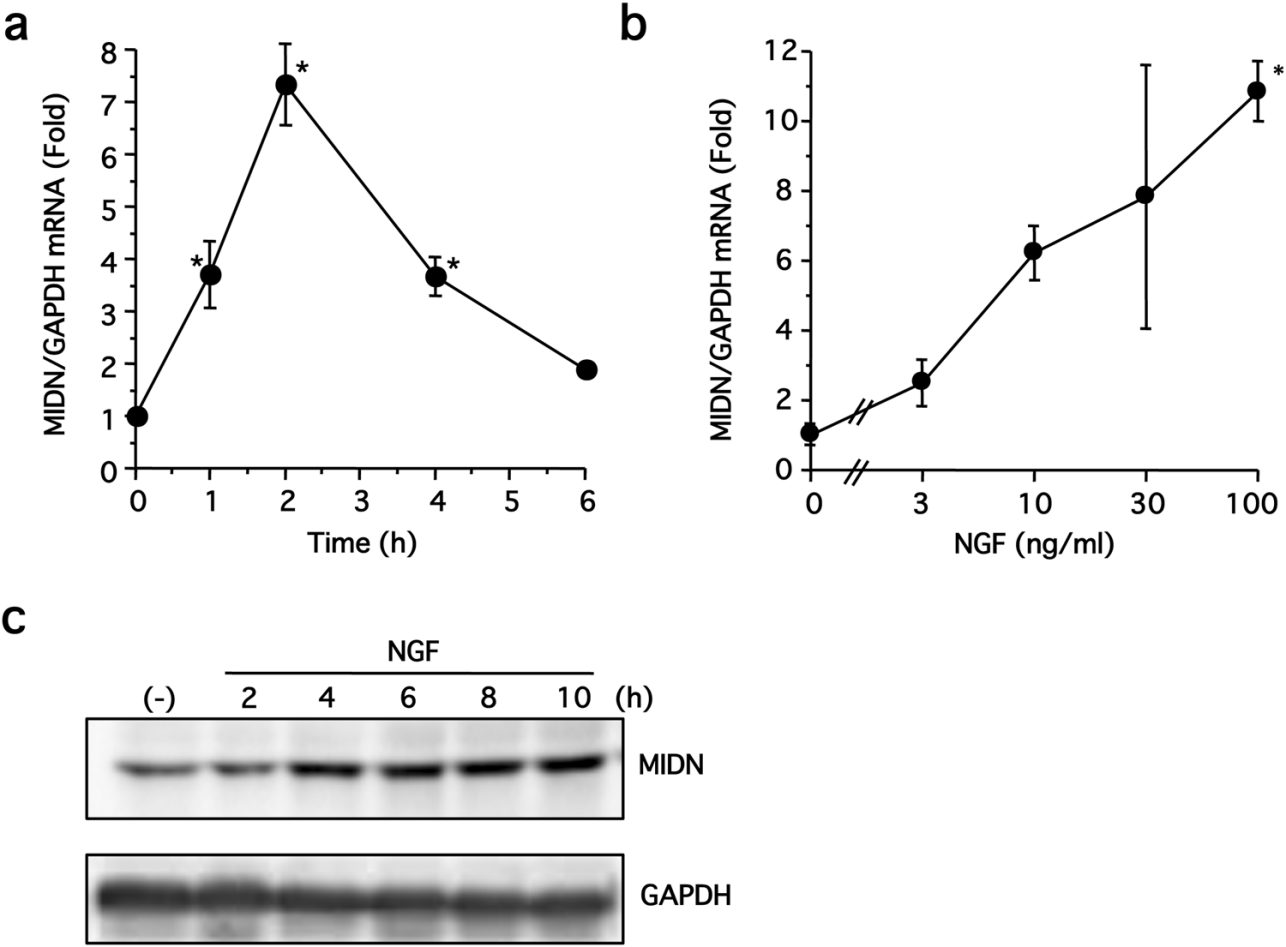
MIDN expression is induced by NGF in PC12 cells. (**a**) Serum-starved PC12 cells were stimulated with 100 ng/ml NGF for 1–6 h, as indicated, then MIDN gene expression was examined by qRT-PCR. NGF significantly induced MIDN gene expression compared with drug-free control (*p < 0.05, n = 3). (**b**) Serum-starved PC12 cells were stimulated with 3–100 ng/ml NGF, as indicated, for 2 h, then MIDN gene expression was examined by qRT-PCR. NGF significantly induced MIDN gene expression compared with drug-free control (*p < 0.05, n = 3). (**c**) Serum-starved PC12 cells were stimulated with 100 ng/ml NGF for 2–10 h, as indicated, and MIDN protein levels were examined by Western blotting.

Firstly, MIDN gene expression in PC12 cells in the presence or absence of NGF was examined by quantitative RT-PCR (qRT-PCR). PC12 cells were stimulated with NGF (100 ng/ml) for 1–6 h or NGF (3–100 ng/ml) for 2 h, then MIDN expression was examined. NGF induced MIDN gene expression in a time- and concentration-dependent manner (Fig. 1a and b). Stimulating the cells with 100 ng/ml NGF for 1–6 h caused a rapid increase in MIDN mRNA expression, peaking after 2 h, before a gradual return to control levels after 6 h (Fig. 1a). Increasing the NGF concentration from 3–100 ng/ml triggered a corresponding increase in MIDN expression measured after 2 h (Fig. 1b). Considering the MIDN protein, levels steadily increased for 4 h after stimulation with 100 ng/ml NGF, and showed no reduction after 10 h, indicating that the protein is stable for at least this length of time (Fig. 1c).

**Figure 2:**
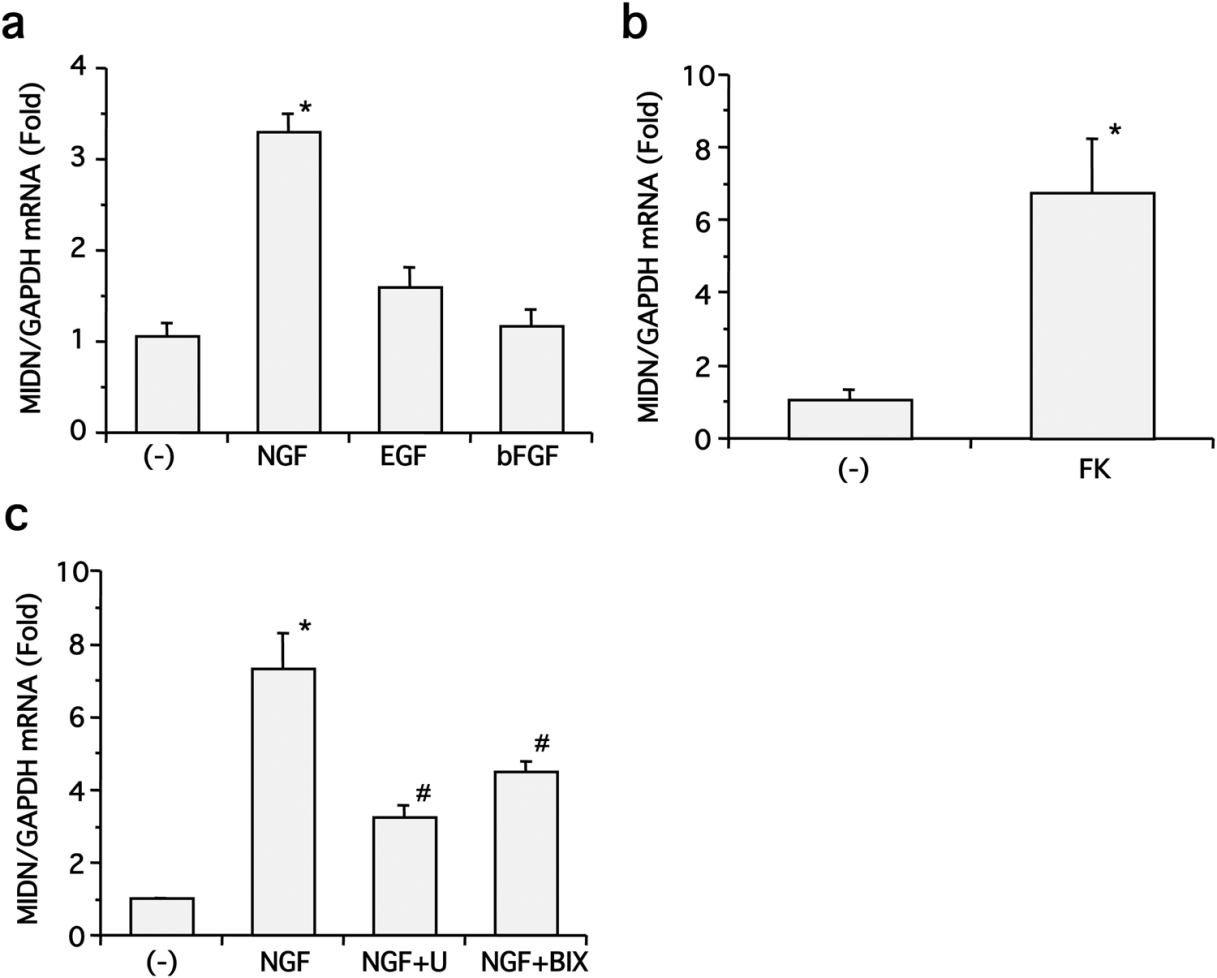
NGF-induced MIDN gene expression is mediated by ERK1/2 and ERK5, and cAMP signaling promotes MIDN gene expression in PC12 cells. (**a**) Serum-starved PC12 cells were stimulated with or without 100 ng/ml of NGF, EGF, or bFGF for 2 h, then MIDN gene expression was examined by qRT-PCR. NGF significantly induced MIDN gene expression compared with drug-free control (*p < 0.05, n = 6). (**b**) Serum-starved PC12 cells were stimulated with or without forskolin (FK, 10 μM) for 2 h, then MIDN gene expression was examined by qRT-PCR. Forskolin significantly induced MIDN gene expression compared with drug-free control (*p < 0.05, n = 3). (**c**) Serum-starved PC12 cells were stimulated with or without 100 ng/ml NGF for 2 h in the presence or absence of U0126 (U, 30 μM) or BIX02189 (BIX, 30 μM), then MIDN gene expression was examined by qRT-PCR. NGF significantly induced MIDN expression compared with drug-free control (*p < 0.05, n = 3), and the induction of MIDN expression by NGF was significantly blocked by either U0126 or BIX02189 (^#^ *p* < 0.05).

To examine whether MIDN expression was controlled by other growth factors, the expression of MIDN mRNA in PC12 cells was also investigated following stimulation with 100 ng/ml of epidermal growth factor (EGF) or basic fibroblast growth factor (bFGF). Interestingly, while NGF significantly promoted MIDN gene expression, this was not seen with EGF or bFGF (Fig. 2a). Next, we examined the effect of pharmacological reagents that modulate either second messenger levels or signaling molecule activity on MIDN gene expression. We found that neither 100 nM of the protein kinase C activator phorbol-12-myristate-13-acetate (PMA) nor 1 μM of the Ca^2+^ ionophore A23187 had any appreciable effect on MIDN gene expression (PMA 1.46 ± 0.175 fold, *p* > 0.05, n = 3; A23187 1.17 ± 0.117 fold, *p* > 0.05, n = 3), but that 10 μM forskolin, an adenylyl cyclase activator, significantly induced MIDN expression (Fig. 2b). This suggests that cAMP signaling regulates MIDN gene expression.

Previously, we showed that NGF strongly activates both ERK1/2 and ERK5 in PC12 cells^6, 7^. ERK1/2 activity is regulated by the small G-proteins Ras and Rap1^8, 9^. On the other hand, ERK5 activity has been reported to be independent of these G-proteins^6^, although the regulation of ERK5 by Ras and Rap1 has been controversial^10^. To investigate this further, we treated PC12 cells with 100 ng/ml NGF for 2 h in the presence or absence of 30 μM U0126 or BIX02189, these being verified specific inhibitors of MEK1/2 and MEK5, respectively^6, 7^. We found that the induction of MIDN expression by NGF was impaired significantly by both inhibitors (Fig. 2c), suggesting that this induction is mediated via ERK1/2 and ERK5. It has been shown that the oncogenic Ras mutant, RasV12, activates ERK1/2 strongly, and that the constitutively active MEK5 mutant, MEK5D, selectively activates ERK5^6, 11^. We therefore overexpressed RasV12 or MEK5D in PC12 cells and examined MIDN gene expression. However, no significant induction of MIDN gene expression was observed, suggesting that ERK activity is necessary but not sufficient for MIDN induction.

**Figure 3:**
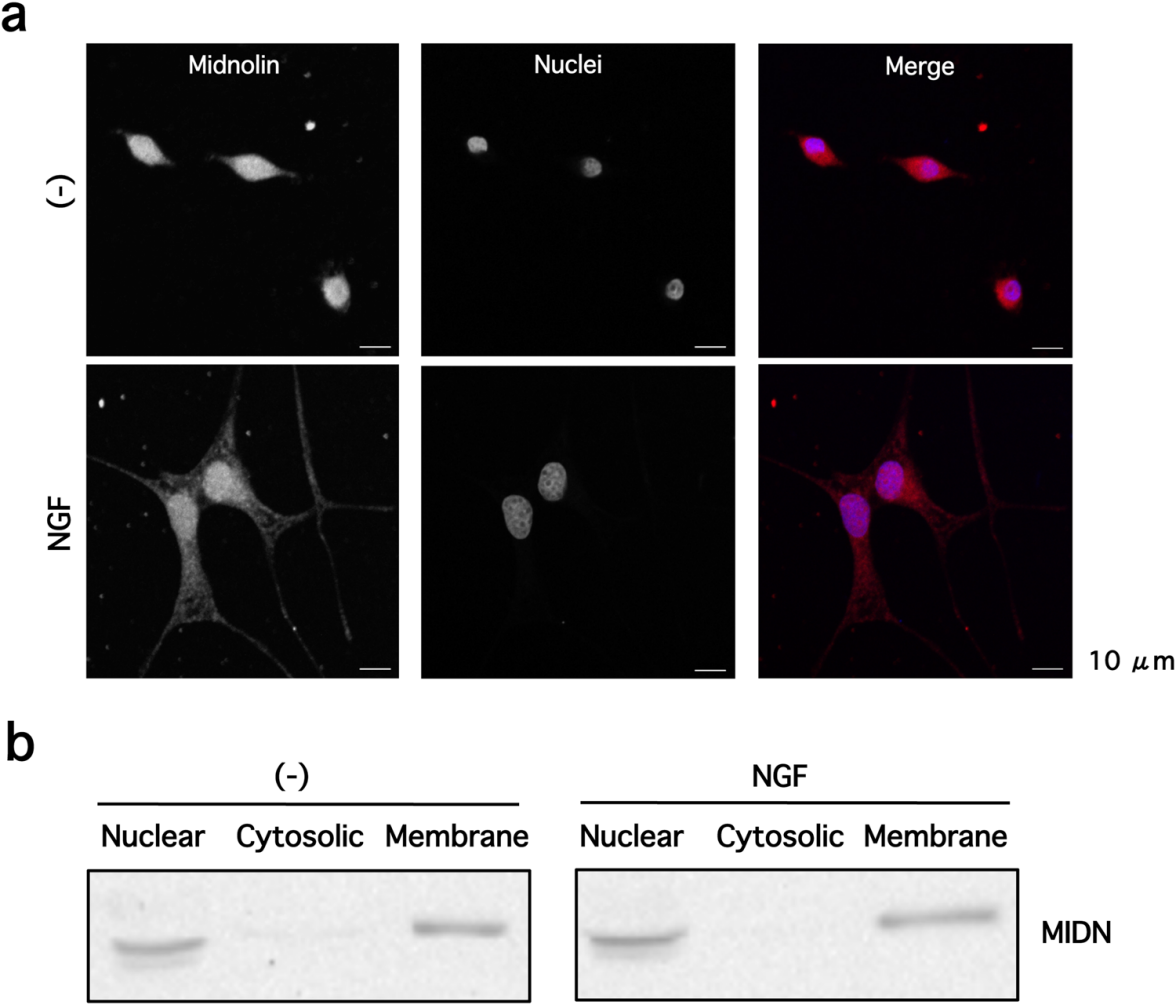
MIDN localizes primarily to the nucleus and intracellular membranes in PC12 cells. (**a**) PC12 cells were incubated in the presence or absence of 100 ng/ml NGF for one day before being stained with anti-MIDN antibody. Localization of endogenous MIDN was observed using a confocal laser scanning microscope. MIDN and nuclei were colored in red and blue, respectively in the merge image. Scale bar: 10 µm **(b)** Following the NGF incubation described above, cells were homogenized and fractionated into nuclear, cytosolic, and crude membrane fractions as described in Methods. The MIDN in these fractions was then examined by Western blotting.

There have been conflicting reports regarding the subcellular localization of endogenous MIDN^1, 3^, so we examined this in PC12 cells. Our immunofluorescence results showed that MIDN was mainly localized to the nucleus and intracellular vesicle membranes in both undifferentiated and differentiated PC12 cells (Fig. 3a). To investigate further, cells were fractionated and intracellular organelles were isolated, then MIDN expression in each fraction was examined by Western blotting. Consistent with the immunofluorescence results, MIDN was clearly visible in both the nuclear and crude membrane fractions, with the latter including the intracellular vesicle membrane fraction, although a faint band was also observed in the cytosolic fraction (Fig. 3b). Again, no difference in subcellular localization was observed following differentiation, which was induced by NGF.

### MIDN is associated with neurite outgrowth and parkin expression, and its loss is associated with sporadic PD

MIDN is strongly expressed in the midbrain^1^, and its ubiquitin-like domain shares homology with that of parkin^3^. Furthermore, ERK5, which is activated by NGF in PC12 cells, is a principal regulator of catecholamine biosynthesis^7^. Having shown that MIDN expression is induced by NGF, and that this induction is modulated by the MAPK pathway, we examined the association between these results and PD. Previously, we reported a genetic association study comparing healthy elderly people and patients with sporadic PD that revealed two functional single nucleotide polymorphisms within G-protein-coupled receptor kinase (GRK) 5 introns in sporadic PD patients. Further, GRK5 was shown to phosphorylate α-synuclein at Ser129, resulting in the formation and aggregation of soluble α-synuclein oligomers^12^. Similarly, GRK6 has been shown to phosphorylate and potently regulate α-synuclein^13^. In the present study, no single nucleotide polymorphisms or copy number variations were observed between the sporadic PD and control groups in ERK5, MEK5, or ankyrin-repeat domain 1 (ankrd1), a protein that is induced by ERK5 and which stabilizes TH^7^. Interestingly, however, we found a significant copy number variation in MIDN, with 10.5% of sporadic PD patients possessing only a single copy of the gene compared with 0% in the control group (Table 1). Of the nine PD patients displaying a reduction in MIDN copy number, a clear gender bias towards women was seen, with the male ratio being 0.11; conversely, the 77 PD patients with no MIDN loss were gender balanced, with a male ratio of 0.44. While these gender differences were not considered statistically significant, the tendency for MIDN gene loss to affect women was clear (p < 0.1). No significant single nucleotide polymorphisms within MIDN gene were discovered in our specimens.

**Table 1:** MIDN copy number analysis in patients with sporadic PD and elderly normal controls.

	MIDN gene			
	single CN		normal CN	
sporadic PD (n = 86)	9	10.5%	77	89.5%
elderly control (n = 100)	0	0%	100	100%

The MIDN gene copy numbers (CN) in 86 sporadic PD patients and 100 elderly controls were analyzed as described in Methods. There was significant MIDN copy number variation between sporadic PD patients and controls, with 10.5% patients possessing only a single copy of the MIDN gene, compared to no gene loss in healthy controls (*p* < 0.01).

**Figure 4:**
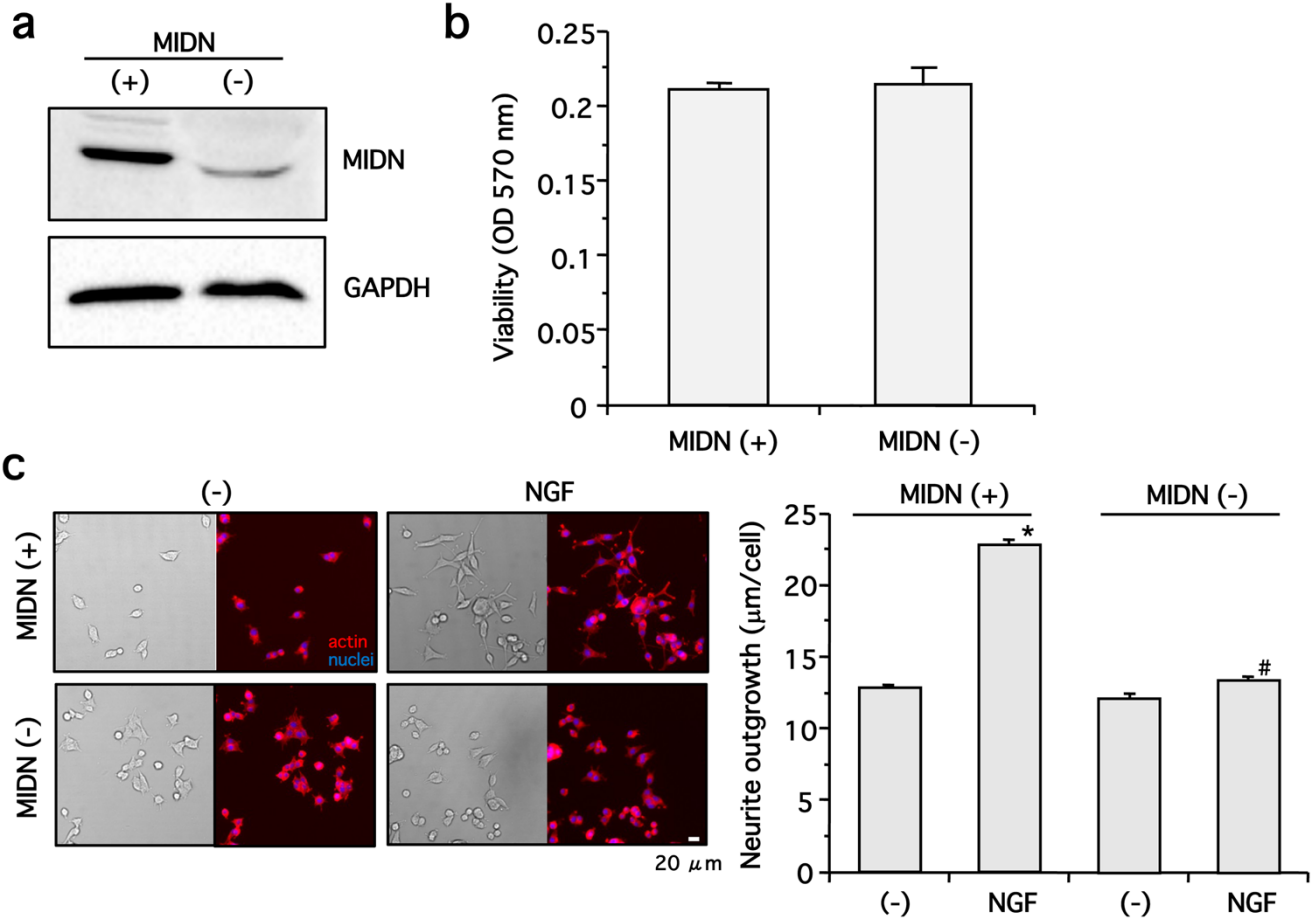
MIDN is necessary for NGF-induced neurite outgrowth in PC12 cells. (**a**) MIDN-positive (+) and -negative (−) PC12 cells were generated using CRISPR/Cas9 technology, then MIDN protein expression was examined by Western blotting. **(b)** The viability of the MIDN-positive (+) and -negative (−) PC12 cells was examined using an MTT assay, measuring absorbance at 570 nm (n = 5). **(c)** The MIDN-positive and -negative PC12 cells were incubated in the presence or absence of 100 ng/ml NGF in serum-free medium for one day. Cells were fixed in 4% paraformaldehyde, and the actin filaments and nuclei were stained with rhodamine-phalloidin (5 U/ml) or Hoechst-33258 (1 μg/ml), respectively. Forty randomly selected areas per well were photographed, then the total neurite length was divided by the number of nuclei to give the neurite length per cell (μm/cell). While NGF significantly promoted neurite outgrowth in MIDN-positive cells compared with drug-free control (*p < 0.05, n = 6), the neurite outgrowth in MIDN-negative cells was attenuated compared with MIDN-positive cells (^#^ *p* < 0.05, n = 6).

Having shown that MIDN gene loss was related to PD, we next examined the roles of MIDN in PD pathogenesis in detail. First, we used CRISPR/Cas9 technology to create MIDN-deficient polyclonal PC12 cells (termed ‘MIDN-negative cells’) as described in the Methods. As expected, MIDN-negative cells displayed lower MIDN protein expression than MIDN-positive cells (Fig. 4a), with this difference having no significant effect on cell viability, as determined by an MTT assay (Fig. 4b). Next, the neurite outgrowth induced by NGF was examined in these cells; while NGF significantly promoted neurite outgrowth in MIDN-positive cells, neurite outgrowth in MIDN-negative cells were attenuated compared with MIDN-positive cells (Fig. 4c).

**Figure 5:**
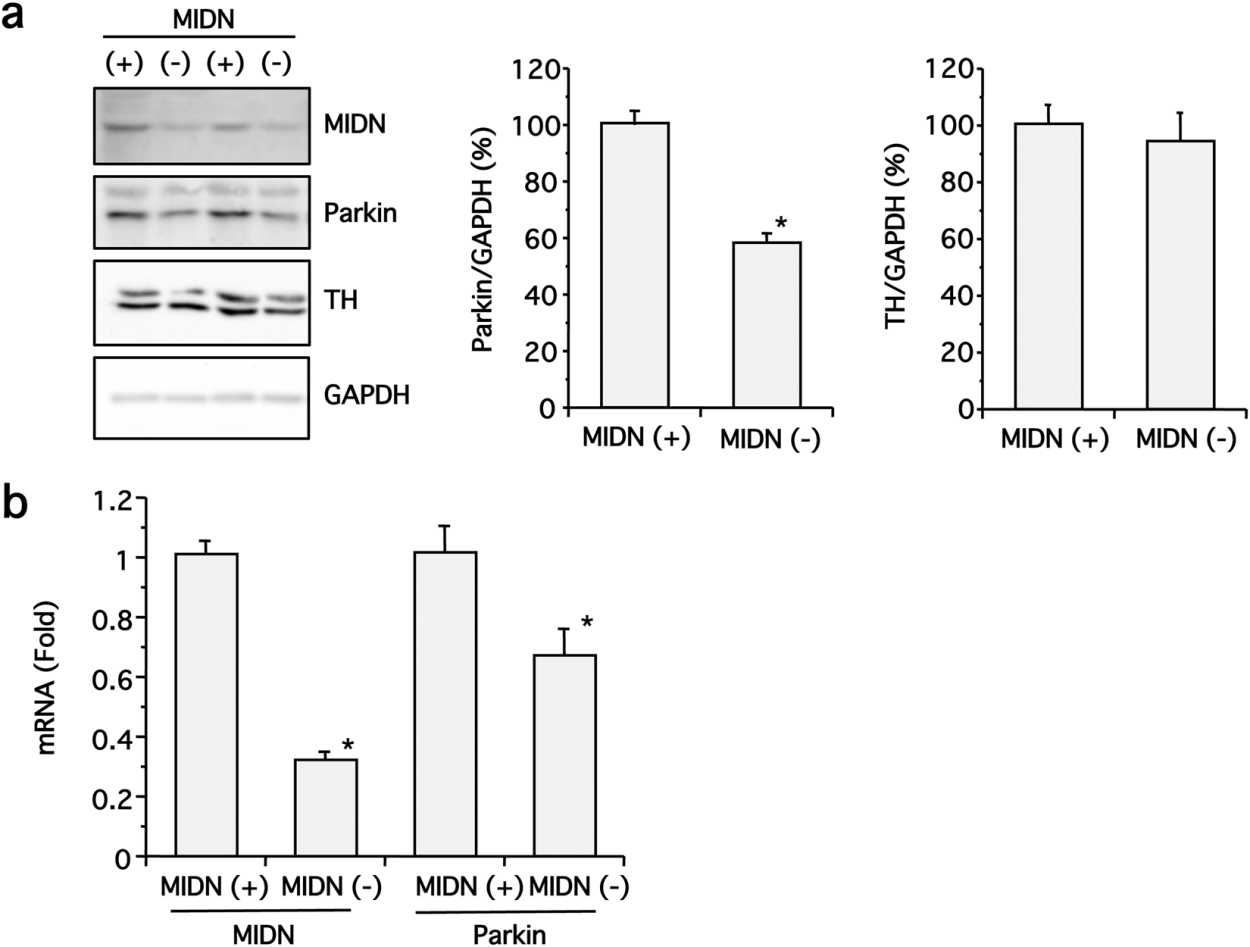
MIDN is necessary for parkin expression in PC12 cells. (**a**) MIDN, parkin, TH, and GAPDH protein levels in MIDN-positive and -negative PC12 cells were examined by Western blotting (left panel). The density of the parkin and TH bands was normalized to that of GAPDH, and these ratios were expressed as a percentage of MIDN-positive cells (middle and right panels). Parkin protein expression was significantly inhibited by MIDN knockout (*p < 0.05, n = 6) whereas TH protein expression was not significantly changed (n = 6). **(b)** Gene expression levels of MIDN and parkin in MIDN-positive and -negative PC12 cells were examined by qRT-PCR. Significant differences in both MIDN and parkin gene expression were observed between MIDN-positive and -negative cells (*p < 0.05, n = 6).

Next, we examined the protein expression of parkin and TH in our MIDN-positive and -negative PC12 cells. While no significant difference in TH levels was observed between the two cell types, parkin expression was reduced by approximately 40% in MIDN-negative cells, a statistically significant reduction (Fig. 5a). A corresponding significant reduction in parkin mRNA expression was observed in MIDN-negative cells, suggesting that MIDN regulates parkin gene expression (Fig. 5b).

**Figure 6:**
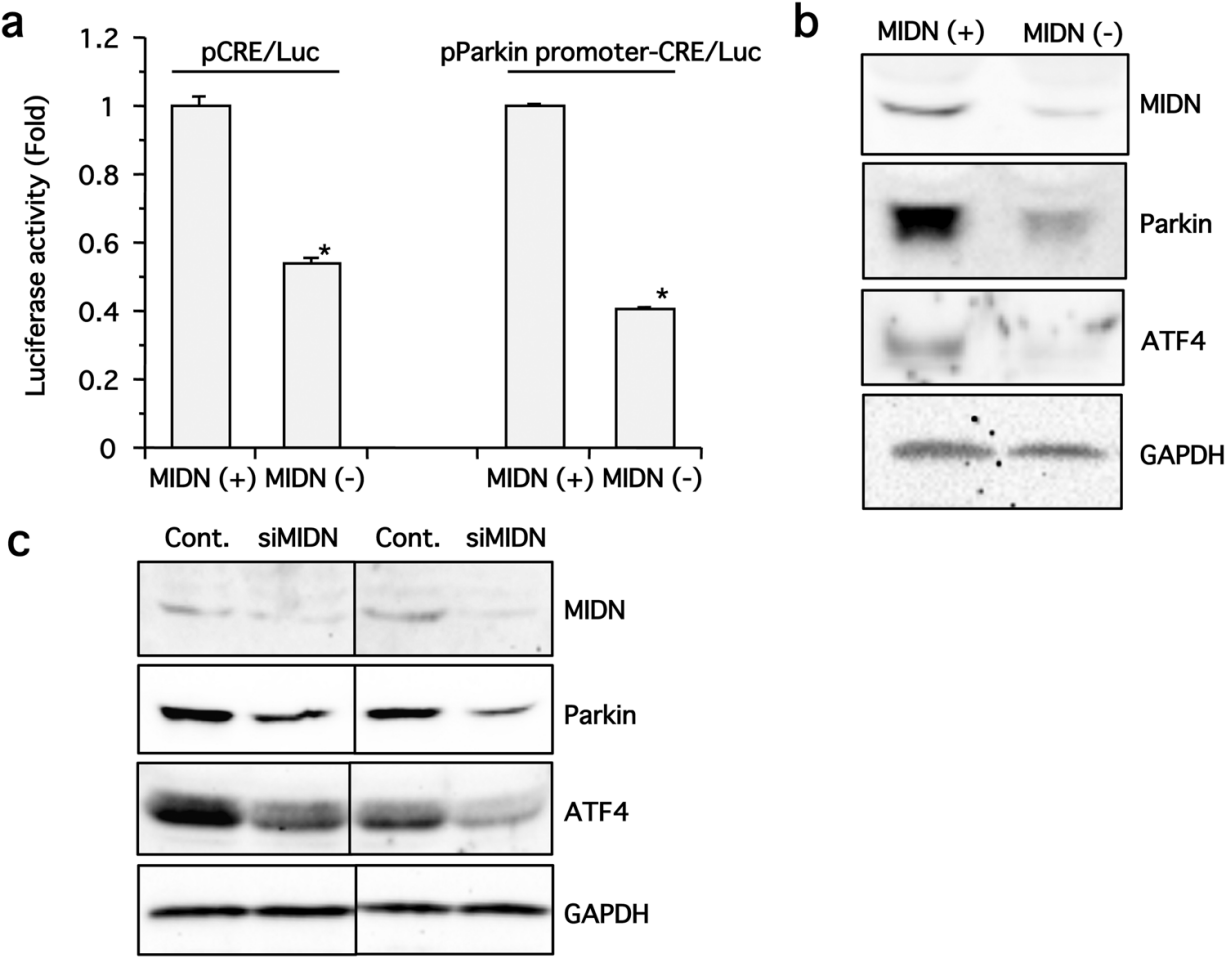
MIDN regulates parkin expression via ATF4 in PC12 cells. (**a**) MIDN-positive and -negative PC12 cells were transfected with DNA plasmids encoding either a typical CRE/luciferase or a human parkin promoter-derived CRE/luciferase, then CRE activity was examined by measuring luciferase activity. In both cases, CRE activity differed significantly between MIDN-positive and -negative cells (*p < 0.05, n = 3). **(b)** MIDN, parkin, ATF4, and GAPDH protein levels in MIDN-positive (+) and -negative (−) PC12 cells were examined by Western blotting. **(c)** PC12 cells were transfected with 150 pmol of either control siRNA or MIDN siRNA and cultured for 3 days, then protein expression levels of MIDN, parkin, ATF4, and GAPDH were examined by Western blotting.

The parkin promoter has been well studied, and the core promoter region is known to contain several transcription factor binding sites^14, 15^. Of these, the cAMP response element (CRE), a binding site for cAMP response element-binding protein/activating transcription factor (CREB/ATF) proteins, has been shown to be essential for parkin transcription. Additionally, ATF4 has been shown to be a critical transcription factor for parkin expression^16, 17^. We therefore used a reporter gene assay to measure CRE activity in MIDN-positive and -negative PC12 cells, by transfecting cells with DNA plasmids encoding either tandem artificial CRE sequences linked to luciferase (Fig. 6a, left panel) or tandem CRE sequences derived from the human parkin promoter linked to luciferase (Fig. 6a, right panel), and measuring basal CRE luciferase activity. CRE activity was significantly lower in MIDN-negative cells than in MIDN-positive cells with both reporter plasmids (Fig. 6a), suggesting that MIDN promotes CRE activity on parkin promoter. Consistent with this, ATF4, which binds to the CRE site to regulate parkin expression, was down-regulated in MIDN-negative cells (Fig. 6b), and both parkin and ATF4 protein levels were reduced when MIDN was knocked down in an siRNA experiment (Fig. 6c), minimizing the possibility of off-target effects accounting for these results. Taken together, these results suggest that MIDN induces ATF4 expression, which in turn induces parkin gene expression via the CRE site in the parkin core promoter.

## Discussion

**Figure 7:**
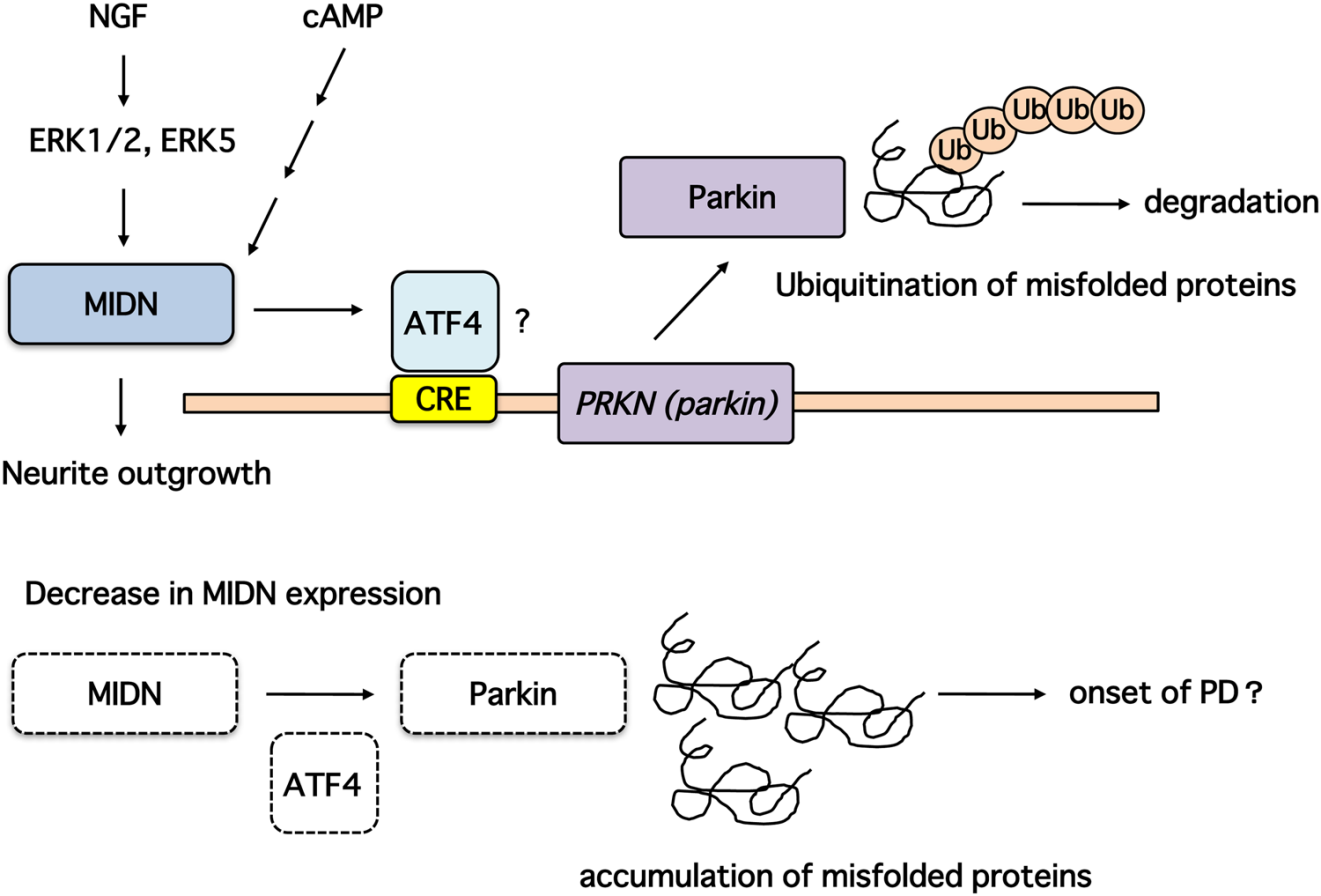
A putative signaling pathway of parkin expression induced by MIDN. Under normal conditions, MIDN is upregulated by both NGF, via ERK1/2 and ERK5, and cAMP signaling. MIDN then promotes neurite outgrowth. Parkin gene expression is promoted by MIDN and ATF4 via the CRE binding site in the parkin core promoter. Parkin then promotes the ubiquitination of misfolded proteins and maintains protein quality. Conversely, when MIDN is down-regulated, ATF4 expression levels also decrease, causing a down-regulation of parkin and a consequent accumulation of misfolded proteins, which can act as a trigger for PD.

In the present study, we demonstrate for the first time that MIDN expression occurs primarily in the nucleus and intracellular vesicle membranes of PC12 cells, and is induced by both NGF and cAMP signaling. When MIDN was knocked out, NGF-induced neurite outgrowth, an indicator of neuronal differentiation, was largely inhibited. Furthermore, MIDN deletion resulted in a loss of expression of parkin, suggesting that MIDN loss participates in the accumulation of misfolded proteins in neurons, where they exert toxic effects. Consistent with this, genetic analysis showed that 10.5% of patients with sporadic PD lacked one copy of the MIDN gene. A scheme of the MIDN signaling pathway is shown in Fig. 7.

MIDN, similar to ankrd1, was initially found to be an ERK5-inducible gene in PC12 cells following microarray analysis^7^. ERK5 is essential for catecholamine biosynthesis in PC12 cells, sympathetic neurons, and human adrenal glands^6, 7^, and so it was expected that ERK5 would be associated with catecholamine-related diseases such as PD. It is therefore surprising that, to date, no single nucleotide polymorphisms or copy number changes have been identified in the ERK5, MEK5, or ankrd1 genes of sporadic PD patients. We found that MIDN gene expression was enhanced by NGF via ERK1/2 and ERK5 (Figs 1 and 2a,c), but not by overexpression of RasV12 and MEK5D mutants, which are strong and selective activators of ERK1/2 and ERK5, respectively, indicating that these ERK isoforms were necessary but not sufficient for the induction of MIDN expression.

Our previous results showed that, in PC12 cells, EGF activated both ERK1/2 and ERK5 while bFGF only activated ERK1/2^6, 7^. The reason why these growth factors did not promote MIDN induction is currently unclear, but it appears that NGF-specific signaling pathways, in addition to those involving ERKs, are required for MIDN induction. Previously, we reported that ERK5 regulates the expression of glial cell-derived neurotrophic factor (GDNF) in C6 glioma cells^18^, but the involvement of GDNF in MIDN expression remains unclear. In future, it will be interesting to examine the impact of both brain-derived neurotrophic factor and GDNF on MIDN expression, as these are neurotrophic factors that act on the dopaminergic neurons in the substantia nigra, and their administration has shown potential in clinical and preclinical trials for the treatment of PD^19^.

We found that forskolin also induces MIDN expression, although the mechanism by which this occurs remains unclear (Fig. 2b). Previously, we showed that cAMP signaling activated ERK1/2 via Rap1 in PC12 cells and via Ras in cerebellar granule neurons, respectively, but did not activate ERK5 in PC12 cells^6, 8, 20^. The results of the current study suggest that cAMP may enhance MIDN expression via the putative CREB/ATF binding motif in the MIDN promoter, although further experiments are required to confirm this.

Our data show that endogenous MIDN is localized to the nucleus and internal vesicle membranes in PC12 cells (Fig. 3), in contrast to a previous report, which observed the strongest MIDN signal in the nucleolus of CHO cells. However, rather than considering the native protein, these researchers used overexpressed, GFP-tagged MIDN, which could account for the observed differences^1^. A separate study showed that MIDN was localized to the nucleus and cytoplasm of pancreatic β-cells^3^, which could be consistent with the faint MIDN band that we observed in the cytosolic fraction of PC12 cells (Fig. 3b). MIDN protein is reportedly highly expressed in a range of tissues, including the pancreatic β-cells, liver, muscle, and brain of adult mice, as well as in cell lines of human and rat origin^3^, and it is possible that the subcellular localization of MIDN may vary among tissues depending on expression levels of MIDN protein.

We have shown, for the first time, that NGF-induced neurite outgrowth was attenuated by the loss of MIDN in PC12 cells (Fig. 4c). In order to promote neural differentiation, NGF regulates the transcription of the necessary genes^21^. We demonstrated previously that ERK1/2 and ERK5 mediated the transcription of those genes^6, 8^. As MIDN is mainly located in the nucleus, it is possible that it is regulated by ERK1/2 and ERK5 before going on to regulate the transcription of genes necessary for neurite outgrowth. However, whether the loss of MIDN from dopaminergic neurons in the midbrain also results in an attenuation of neurite outgrowth into the striatum must be confirmed.

Genetic analysis of patients with sporadic PD and healthy controls revealed that, while no MIDN gene copy number variation was observed in controls, a MIDN copy number reduction was observed in 10.5% of sporadic PD patients (Table 1). Furthermore, even where MIDN copy number appeared normal in other PD patients, it is possible that MIDN gene expression was suppressed by epigenetic mechanisms. Because MIDN gene loss was not observed in healthy people, it is possible that the MIDN gene could be a causative factor in familial PD, wherein *de novo* mutagenesis of the MIDN gene in the patient has been transferred to their descendants who are, perhaps, too young to have developed PD symptoms. On the other hand, if similar MIDN mutations are discovered in healthy people, the MIDN gene could be considered a risk factor for the onset of PD rather than as a familial PD gene. So, continuous and wider research is required.

In the present study, we used both CRISPR/Cas9 and RNAi methods to provide the first demonstration that loss of MIDN down-regulates parkin (Figs 5 and 6). It is likely that the reduction in parkin protein resulted from a reduction in gene expression rather than protein modification by post-translational mechanisms. In resting cells, parkin is usually located in the cytosol where most of its substrate is to be found, but it can translocate to dysfunctional mitochondria in response to a loss of mitochondrial membrane potential, or to the endoplasmic reticulum (ER) in response to ER stress, wherein it ubiquitinates targeting misfolded proteins^5, 22, 23^. Conversely, MIDN is located primarily in the nucleus, meaning that MIDN and parkin are unlikely to interact directly. Parkin mRNA expression was significantly reduced on deletion of MIDN (Fig. 5b), although it should be noted that MIDN knockout was not 100%, and MIDN mRNA and protein were still observed in MIDN-negative cells (Fig. 5). Improving the efficiency of MIDN knockout would be expected to amplify the observed reduction in parkin gene expression, although it remains possible that other mechanisms are responsible for down-regulation of parkin. Because MIDN mainly localized in nucleus, it is considered that MIDN affected gene expression as a functional transcription factor. Indeed, it appears that MIDN promotes parkin gene expression via CRE (Fig. 6)^16^. It should be noted, however, that while MIDN has been classified as a transcription factor^2^, it does not possess the common DNA binding domains such as the leucine zipper motif or a general activating domain as seen in other transcription factors. It is therefore possible that, in the cell, MIDN interacts with other transcription factors to modulate their transcriptional activity.

Expression of ATF4, a member of CREB/ATF family, has been shown to be induced in response to neuronal toxin or ER stress, and elevated phospho-eIF2α levels have been shown to activate ATF4^17, 24^. However, it is unclear whether ATF4 is beneficial or toxic in neuronal cells^17^. ATF4-null murine stroke-prone model systems displayed less neuronal loss than controls^25^, the ectopic expression of ATF4 increased the sensitivity of murine cortical neurons to ER stress-induced apoptosis, and neurons lacking ATF4 displayed markedly reduced cell death^26^. Conversely, ATF4 has a central role in resistance against oxidative stress^27^, and ATF4 has been shown to bind directly to the parkin promoter, thus increasing parkin expression in order to overcome ER or mitochondrial stress induced by pharmacological reagents such as thapsigargin, tunicamycin, or carbonyl cyanide m-chlorophenylhydrazone. Additionally, mutating the parkin promoter’s CRE sequence, using a dominant-negative ATF4 mutant, and silencing ATF4 using siRNAs were all shown to impair parkin expression^16^. Furthermore, in PC12 cells and cultured murine ventral midbrain dopaminergic neurons, ATF4 has been shown to confer protection against the dopaminergic neuronal toxins 6-hydroxydopamine and 1-methyl-4-phenylpyridinium by maintaining parkin levels^17^.

In the current study, we have shown that MIDN activates the CRE sequence that ATF4 binds to promote parkin gene expression, and that MIDN knockout or knockdown down-regulates the protein expression of ATF4 and parkin (Fig. 6). However, many genes are expected to be regulated by the CREB/ATF family members that bind to CRE sequence, so it will be important to examine the expression and activity of other familial PD genes, including α-synuclein and PTEN-induced putative kinase 1. A comprehensive analysis of gene expression by microarray or RNA-Seq would be most appropriate to discover the effect of MIDN on other PD-related genes. Furthermore, it will be essential to determine whether PD-like disease is triggered in MIDN knockout mice. Interestingly, an examination of parkin knockout mice revealed no accumulation of parkin substrates and normal dopaminergic neurons within the substantia nigra^28–30^, indicating that additional pathogenic events, such as the overexpression of parkin-associated endothelin receptor-like receptor, are required for neuronal degeneration in these genetically modified animals^28^. If these additional pathogenic events are regulated by MIDN as well as parkin, MIDN knockout mice would be expected to display dopaminergic neuron loss. However, the interpretation of PD-related experimental data from murine systems and its extrapolation to human PD patients is complicated by the substantial difference in lifespan between humans and mice; PD symptoms take several decades to emerge in patients, and the murine lifespan is too short to observe these events.

In conclusion, we present the first report of the physiological and pathological roles of MIDN in neuronal cells. We show that MIDN is involved in neurite outgrowth induced by NGF, and that MIDN is associated with PD, with sporadic PD patients showing significant variation in MIDN copy number. Furthermore, MIDN affects ATF4 expression and promotes parkin expression via the CRE binding site in the parkin core promoter. Additionally, loss of MIDN caused the down-regulation of parkin, which could potentially trigger PD. Future experiments should further examine the role of MIDN *in vitro* as well as the characteristics of MIDN-knockout mice.

## Methods

### Materials

NGF, EGF, bFGF, and Hoechst-33258 were purchased from Sigma-Aldrich (St. Louis, MO). The MEK1/2 inhibitor U0126, primary anti-parkin (#4211), anti-glyceraldehyde-3-phosphate dehydrogenase (GAPDH) (#2118), anti-TH (#2792), and anti-ATF4 (#11815) antibodies, as well as horseradish peroxidase (HRP)-conjugated anti-rabbit IgG secondary antibody (#7074) and HRP-conjugated anti-GAPDH antibody (#3683) were purchased from Cell Signaling Technology (Beverly, MA). Anti-MIDN antibody (#251273) was purchased from Abbiotec (San Diego, CA) which has been characterized for specificity^3^, and HRP-conjugated anti-mouse IgG secondary antibody (#NA931) was purchased from GE Healthcare (Buckinghamshire, England). Enhanced chemiluminescence (ECL) assay kits were purchased from either GE Healthcare or PerkinElmer (Waltham, MA). Lipofectamine 2000, Alexa 633-conjugated anti-rabbit IgG antibody, and rhodamine-phalloidin were purchased from Invitrogen (Grand Island, NY). PMA and forskolin were purchased from Wako Pure Chemicals (Tokyo, Japan), A23187 was purchased from Calbiochem (San Diego, CA), and MTT was purchased from Dojindo (Kumamoto, Japan). TriPure Isolation Reagent for total RNA extraction, and the FastStart Essential DNA Green Master for real-time PCR were purchased from Roche (Indianapolis, IN), and a Reverse Transcription kit was purchased from Toyobo (Osaka, Japan). For genome editing experiments, a DNA plasmid encoding Cas9, GFP, and a gRNA with the sequence 5′-CGT GCC ACA CGC TGC AAA GTG G-3′, targeting MIDN, was designed by and purchased from Sigma-Aldrich. Control siRNA was purchased from B-Bridge (Mountain View, CA), and siRNAs targeting rat MIDN were synthesized by Invitrogen. When targeting MIDN in siRNA experiments, a cocktail of three duplexes was used as follows: 1) sense 5′-ACU CCG UUU GGA AGC CUG AAG UCA A-3′ and antisense 5′-UUG ACU UCA GGC UUC CAA ACG GAG U-3′; 2) sense 5′-CAA UCC CAU GCA GCC UCC ACG ACU U-3′ and antisense 5′-AAG UCG UGG AGG CUG CAU GGG AUU G-3′; and 3) sense 5′-CAG GGA CCU UCU CUG GCA CAC UGC A-3′ and antisense 5′-UGC AGU GUG CCA GAG AAG GUC CCU G-3′. A DNA plasmid encoding a tandem CRE site-driven firefly luciferase was purchased from Takara (Tokyo, Japan), and an additional plasmid encoding firefly luciferase driven by a tandem CRE site derived from the human parkin promoter was kindly provided by Dr. Konstanze F. Winklhofer (Ruhr University Bochum, Germany)^16^. The MEK5/ERK inhibitor BIX02189 was kindly provided by Boehringer Ingelheim (Ridgefield, CT).

### Cell Culture

PC12 cells were grown in Dulbecco’s Modified Eagle’s medium (DMEM) supplemented with 10% heat-inactivated fetal calf serum, 5% horse serum, 50 units/ml penicillin, and 50 μg/ml streptomycin, in a 37 °C incubator containing 5% CO_2_. In gene editing experiments, the cells were transfected with a CRISPR/Cas9-DNA plasmid with a gRNA targeting MIDN, and then GFP-negative and -positive cells, indicating MIDN-positive and -negative cells, respectively, were isolated using a FACSAria cell sorter (BD Biosciences Japan, Tokyo, Japan) as described previously^6^. MIDN-positive and -negative cells were cultured as described above. The transfection and cell sorting procedure was repeated, and the sorted cells were then used in the experiments described in Figs 4c, 5b and 6.

### Assay for Neurite Outgrowth

The extension of neurites from PC12 cells is recognized as an index of neuronal differentiation. To assay neurite outgrowth, cells were fixed in 4% paraformaldehyde before actin filaments and nuclei were stained with rhodamine-phalloidin (5 U/ml) and Hoechst-33258 (1 μg/ml), respectively. Cellular images were then obtained using a Cellomics ArrayScan XTI imaging platform (ThermoFisher Scientific KK, Tokyo, Japan). Analysis of the number of nuclei and the total length of neurites was performed using the HCS Studio Neuronal Profiling BioApplication V4.2 (ThermoFisher Scientific KK), before the neurite length per cell (μm/cell) was calculated by dividing the total neurite length by the number of nuclei, as described previously^6^.

### Sodium Dodecyl Sulfate-Polyacrylamide Gel Electrophoresis and Western Blotting

Proteins were separated by electrophoresis using 11% polyacrylamide gels. Proteins were then transferred from the gel onto a polyvinylidene difluoride membrane (GE Healthcare) using a semi-dry blotting method according to standard protocols. Membranes were blocked for 0.5 h at room temperature in 5% skim milk in Tris-buffered saline containing 0.1% tween-20 (TBST), then incubated with the indicated primary antibodies overnight at 4 °C. Antibodies were dissolved in the blocking buffer described above, and used at the following dilutions: anti-MIDN (1:500), anti-GAPDH (1:1000), anti-parkin (1:1000), anti-TH (1:1000), and anti-ATF4 (1:1000). The membranes were washed several times with TBST before being incubated with HRP-conjugated anti-rabbit or anti–mouse IgG secondary antibodies (diluted 1:5000 in blocking buffer) at room temperature for 2 h. Membranes were then washed with TBST, developed using an ECL chemiluminescence assay kit, and visualized using a ChemiDoc XRS imaging system (BioRad, Hercules, CA). The relative intensities of the bands corresponding to parkin and the internal control GAPDH were determined using Image-J densitometry software.

### qRT-PCR

Total RNA from PC12 cells was extracted using TriPure isolation reagent according to the manufacturer’s protocol. RNA was then reverse transcribed using an RT-PCR kit, and real-time PCR was performed using a LightCycler Nano thermal cycler (Roche), as described previously^7^. The PCR primers used in PC12 cell experiments were as follows: MIDN (5′-CCC CAA CTG CCA GGA TAG TA-3′ and 5′-GGT AGT TTT GGG GGT GAG GT-3′), GAPDH (5′-ACC ACA GTC CAT GCC ATC AC-3′ and 5′-TCC ACC ACC CTG TTG CTG TA-3′), and parkin (5′-CTT CCA GCT CAA GGA AGT GG-3′ and 5′-CAG AGG CAT TTG TTT CGT GA-3′). The PCR primers used to confirm the efficiency of MIDN deletion were as follows: 5′-GTG CCA CAC GCT GCA AAG-3′ and 5′-AAC TCG GAC TGG ATG TCT GG-3′. PCR products were quantified and normalized to the GAPDH control before finally being presented as a fold change.

### Immunofluorescence

PC12 cells were fixed in 4% paraformaldehyde and then permeabilized with 0.5% Triton X-100 in phosphate-buffered saline (PBS) for 5 min, before being incubated in 5% bovine serum albumin (BSA)/PBS at 37 °C for 1 h. The fixed cells were then stained sequentially with an anti-MIDN primary antibody (diluted 1:500 in 1% BSA/PBS) at 4 °C overnight, then an Alexa 633-conjugated anti-rabbit IgG secondary antibody (diluted 1:200 in 1% BSA/PBS) at room temperature for 1 h. Nuclei were stained with 1 μg/ml Hoechst-33258. Finally, cells were imaged using a LSM-700 confocal laser scanning microscope (Carl Zeiss, Oberkochen, Germany).

### Subcellular Fractionation

Cells were incubated for one day with or without NGF, then homogenized in buffer A (10 mM Hepes pH 7.3, 0.3 M sucrose, 1 mM phenylmethylsulfonyl fluoride, 10 μg/ml leupeptin, 10 μg/ml aprotinin, and 1.5 mM Na_3_VO_4_). The homogenates were then centrifuged at 800 × *g* at 4 °C for 10 min, the supernatants (S1) were aspirated, and the pellets (P1) were dissolved in Laemmli buffer and designated the nuclear fraction. The S1 fractions were then centrifuged at 100,000 × *g* at 4 °C for 30 min, and a second supernatant (S2) was aspirated and designated the cytosolic fraction. The second pellet (P2) was then lysed in buffer B (50 mM Tris-HCl pH 7.5, 1 mM EDTA, 1 mM EGTA, 0.5 mM Na_3_VO_4_, 0.1% 2-mercaptoethanol, 10 mM glycerophosphate, 0.1 mM phenylmethylsulfonyl fluoride, 1% Triton X-100, 50 mM NaF, and 5 mM Na_2_HPO_4_), and was centrifuged at 100,000 × *g* at 4 °C for 30 min. The resulting supernatant (S3) was dissolved in Laemmli buffer and designated the crude membrane fraction. All fractions dissolved in Laemmli buffer were then boiled at 95 °C for 5 min, as described previously^7, 31^.

### Reporter Gene Assays

Reporter gene assays were performed similarly as described previously^6^. CRE-luciferase reporter genes were introduced using the plasmids described above. The reporter sequences encoded by the commercial pCRE-luciferase plasmid and the human parkin promoter-derived CRE-luciferase plasmid were TGACGTCA-TGACGTCT-TGACGTCA-luciferase and TGACGTAA-TGACGTAA-TGACGTAA-luciferase, respectively. PC12 cells were seeded at a density of 1 × 10^5^ cells/well in 24-well plates and cultivated for one day. One microgram of reporter plasmid and 1 μl/tube of the transfection reagent Lipofectamine 2000 were mixed gently with DMEM to a final volume of 10 μl/tube, the mixture was incubated at room temperature for 20 min, and then a further 40 μl/tube DMEM was added. Next, the cell medium was replaced with 200 μl/well serum-free DMEM, and the entire 50 μl of transfection mixture was transferred to each well. Cells were then incubated at 37 °C for 4–6 h before the medium was replaced with 500 μl/well of growth medium supplemented with 10% fetal calf serum and 5% horse serum. To assess luciferase activity, cells were lysed in 100 μl/well lysis buffer (1% Triton X-100, 110 mM K_2_HPO_4_, 15 mM KH_2_PO_4_ (pH 7.8)), then lysates were centrifuged to remove cellular debris, and 50 μl of the supernatant was mixed with 300 μl of assay buffer (25 mM Gly-Gly, 15 mM MgSO_4_, 5 mM ATP, 10 mM NaOH). The luciferase reaction was then initiated with 100 μl of 150 μM luciferin solution, and luciferase activity was measured using a GloMax 20/20 luminometer (Promega, Madison, WI).

### MTT Assay

MIDN-positive and -negative PC12 cells were seeded at a density of 5 × 10^4^ cells/well in 96-well plates and cultured for two days. Next, 0.1 mg/well MTT was added to the cells, and the plates were incubated for a further 2 h at 37 °C before the culture medium was replaced with dimethyl sulfoxide. Viability was assessed by measuring the absorbance of reduced MTT at 570 nm using a Sunrise plate reader (Tecan Japan, Kawasaki, Japan).

### Subjects

Patients with sporadic PD were enrolled through the Yamagata Cohort Study, which surveyed all of the 782 hospitals and clinics located in the Yamagata prefecture in northwest Japan. Eighty-six L-dopa-responsive sporadic PD patients were enrolled with a mean age of onset of 64.7 ± 7.2 years, and a male ratio of 0.41. The PD diagnosis was made by neurologists and neurosurgeons after comprehensive interviews and medical and neurological examinations. One hundred sex-matched elderly control participants with an age range of 70–71 and a male ratio of 0.41 who displayed no apparent neurological symptoms were recruited through the Takahata Cohort Study, a genetic association study taking place in southern Yamagata. The present study was performed in accordance with a protocol approved by the Ethics Committee of Yamagata University (approval no’s 27 and 61). Written informed consent was obtained from all study participants. There was no significant difference in gender ratio between the sporadic PD and elderly control groups (Chi-square test, p = 1). Genomic DNA (gDNA) was extracted from peripheral blood mononuclear cells from all participants, then 2 μg gDNA was used to assess gene copy number variations with a Human 370 K SNP-CNV BeadArray microarray (Illumina Inc., CA). Estimated copy number was calculated by Hidden Markov Model algorithm using unix-based PennCNV program^32^ from both the B-allele frequency data and the normalized signal intensity data, which were designed to target the frequently single nucleotide polymorphic regions and the copy number regions.

### Statistics

Data are expressed as means ± S.E.M., and the statistical significance of the differences between groups were analyzed using Student’s t-test, the Wilcoxon rank sum test, Chi-square test and one-way ANOVA with post hoc test using Tukey’s or Dunnett’s tests for multiple comparisons.

### Data availability

The datasets analyzed during the current study are not publicly available due to ethical reasons to protect research participant privacy, but is available from the corresponding author on reasonable request and with a brief written agreement that the requestor does not provide any third party with it. Except the human genomic datasets, all data generated or analyzed during this study are included in this published article.
